# The effect of maternal immunity on the equine gammaherpesvirus type 2 and 5 viral load and antibody response

**DOI:** 10.1371/journal.pone.0218576

**Published:** 2019-06-21

**Authors:** Lilja Thorsteinsdóttir, Sigríður Jónsdóttir, Sara Björk Stefánsdóttir, Valgerður Andrésdóttir, Bettina Wagner, Eliane Marti, Sigurbjörg Torsteinsdóttir, Vilhjálmur Svansson

**Affiliations:** 1 Institute for Experimental Pathology, Biomedical Center, University of Iceland, Keldur, Reykjavík, Iceland; 2 Department of Clinical Research and Veterinary Public Health, Vetsuisse Faculty, University of Berne, Berne, Switzerland; 3 Department of Population Medicine & Diagnostic Sciences, College of Veterinary Medicine, Cornell University, Ithaca, NY, United States of America; University of Hong Kong, HONG KONG

## Abstract

Two types of gammaherpesviruses (γEHV) are known to infect horses, EHV-2 and EHV-5. Foals become infected early in life, probably via the upper respiratory tract, despite maternal antibodies. In this study, we analyzed samples from a herd of mares and their foals. The foals were followed from birth to 22 months of age and the dams during the first 6 months postpartum. Blood and nasal swab samples were taken regularly for evaluation of antibody responses, virus isolation and viral load by qPCR. EHV-2 was isolated on day 5, and EHV-5 on day 12, earlier than previously reported. γEHV specific antibodies were not detectable in serum of foals before colostrum intake but peaked a few days after colostrum. Overall, EHV-2 viral load peaked in nasal swab at three to four months of age, paralleled with decline in maternal antibodies, but EHV-5 viral load did not peak until month 12. Maternal antibodies had a notable effect on the viral load and induction of endogenous antibody production. Foals were grouped in two groups depending on the mare’s γEHV specific total IgG levels in serum at birth, group-high and group-low. Group-high had higher levels of maternal γEHV specific total IgG and IgG4/7 for the first 3 months, but when the endogenous production had superseded maternal antibodies, group-low was higher. The maternal antibodies had an effect on the γEHV viral load. Group-low peaked in EHV-2 viral load one month earlier than group-high. These effects were more evident for EHV-5, as there were seven months between the viral load peaks for the groups. The study provides information on how maternal antibody transfer affects γEHV shedding and antibody production in offspring. It also extends our knowledge on the occurrence of EHV-2 and EHV-5 infection in foals during the first two years of life.

## Introduction

Two gammaherpesviruses are known in horses, equid herpesvirus (EHV) 2 and 5. These viruses are closely related and result in strong serological cross reactivity [1, 2]. Both viruses can coexist in the same horse [3–7] and horses can be simultaneously infected with multiple strains [8, 9]. Foals become infected early in life with both viruses, despite presence of maternal antibodies [10, 11]. EHV-2 infection occurs soon after birth and the majority of foals are infected at the age of 2–4 months, when maternal antibodies decline (reviewed in [12]). EHV-5 infection occurs later [5, 13]. Transmission is horizontal; primary infection occurs probably via the upper respiratory tract from mare to foal [14–16] with more strain variation seen in older foals, possibly due to contact with other horses [3, 8]. EHV-2 and EHV-5 viral load is higher in younger horses compared to adults [17–19]. In adult horses, the viral load of EHV-2 and EHV-5 in nasal shedding varies both within and between individuals [20]. Both viruses can be detected at various sites from diseased and healthy horses at all ages. EHV-2 and EHV-5 are typically present in peripheral blood leukocytes and respiratory secretions [3, 21, 22]. Both viruses are ubiquitous and have been associated with a wide range of clinical signs. EHV-2 has been implicated with keratoconjunctivitis [23–25], mild respiratory disease [13, 22, 26], pneumonia [27, 28], pharyngitis [29] and poor performance [30], and EHV-5 with equine multinodular pulmonary fibrosis [31–33] and dermatitis [34].

Herpesviruses are characterized by latent infection with different sites of lytic and latent infection depending on the virus [35]. The acute infection is in permissive cells. In latency the viral DNA is maintained as a circular episome in the host cell nucleus and B lymphocytes are believed to be the host cells for EHV-2 latency [36]. We have previously shown that in contrast to EHV-2, the detection rate of EHV-5 was unaffected in lymphopenic horses, indicating that EHV-5 could have different target cells from EHV-2 [7]. Mekuria et al., 2017 concluded that B lymphocytes are the major site of latency for both viruses [37]. A recent study, however, showed that both B and T lymphocytes support EHV-5 replication, but replication was not found in monocytes [38].

It is still unclear whether EHV-2 and EHV-5 are capable of crossing the placenta. Both viruses have been detected in aborted fetuses and in the placenta [39–41]. The placenta is a highly vascular organ, and it is possible that the amplified viral DNA was derived from maternal blood. It is also not yet known if EHV-2 and EHV-5 can be transmitted with colostrum or vaginal secretion [15, 30].

Immunoglobulin G (IgG) is the major immunoglobulin class in horse serum and colostrum [42, 43]. Horses have seven IgG subclasses, IgG1 to IgG7 [44, 45]. IgG4/7 and IgG1 are important in protection against intracellular infection [46–48] and IgG3/5 is effective against extracellular pathogens [49]. The epitheliochorial structure of the equine placenta prevents transfer of maternal immunoglobulins to the fetus. After birth, the neonate foal absorbs maternal immunoglobulins from the colostrum. The highest absorption capacity of the neonatal gut immunoglobulins is around 6–8 hours; it declines afterwards and, ends by 24 to 36 hours of age [50–52]. The IgG composition of the foal serum after colostrum intake reflects the mare’s colostrum [53]. The maternal immunoglobulin concentration in the foal serum declines steadily during the following weeks and reaches a minimum at different time points depending on the isotype, the antibody status of the mother and the colostrum intake. The subsequent immunoglobulin increase represents endogenous production in the foal and differs for IgG subclasses (review in [53]). The equine fetus produces IgG1 and the production continues after birth [42]. Endogenous IgG3/5 is detectable in foal serum within the first 5–8 weeks, IgG4/7 by weeks 16–20 [54] and IgE 6–11 months after birth [55, 56]. We wanted to investigate γEHV infection and the interaction with the specific antibodies both maternal and endogenous.

## Materials and methods

### Description of the cohort

Thirty healthy Icelandic horses were used in the study, 15 mares (median age 7 years, range 4–12 years) and their foals, all sired by the same stallion. The mares were kept at the Institute for Experimental Pathology, Keldur, Reykjavík, Iceland, from February 2011 without contact with other horses. All mares foaled in the period from 17 May to 23 June 2011. In July the horses were transported to a farm in North Iceland and kept in pasture together with the same stallion as the year before until September. After September, the horses were in contact with other horses of all ages. In January 2012 foals were weaned and they stayed on the farm until March 2013 when they were transported to Keldur and the last samples were taken immediately after transport.

The horses were maintained according to the Icelandic animal care guidelines for horses (910/2014) with the formal approval from the Icelandic Ethical Committee on Animal Research, license 2011-04-15. All horses were on pasture from May to December and hay-fed outdoors during winter. Throughout the study the horses were regularly observed for health status. They were dewormed annually in the fall, with 0.2 mg of Ivermectin (Merial) per kg of body weight.

### Samples

Blood and nasal swab (NS) samples were collected from the mares and their newborn foals postpartum, before colostrum uptake (D0) and then regularly for 22 months from the foals and for 6 months from the mares (S1 Table).

Blood was collected by jugular venipuncture into EDTA and serum vacutainer tubes (Vacuette, Greiner Bio-One). Leukocytes were isolated as followed: First, red blood cells were allowed to sediment in EDTA tubes for 30 min for mares and 90 min for foals at RT, then 100 μl of the enriched plasma, containing peripheral blood leukocytes (PBL) was used directly for virus isolation. The tubes were then centrifuged, and the buffy coat (BC) layer was harvested and DNA extracted for qPCR.

Nasal swab pins (FLOQSwabs, Coban) were introduced 15 cm via the ventral nasal meatus to collect samples from the nasal mucosa and placed immediately into 3mL UTM-RT transport medium (Coban). Tubes were vortexed, centrifuged, and the supernatants were used directly for virus isolation and DNA extraction for qPCR.

### γEHV specific ELISA

γEHV specific total IgG, IgG1, IgG4/7, IgG5 and IgE levels were measured by ELISA in serum from foals and mares, at days 0, 5 and 12, and at months 1, 2, 3, 4, 5 and 6 for foals and mares and at months 9, 12, 15, 20 and 22 for foals (S1 Table).

The EHV-2 and EHV-5 antigens used in the ELISA were made according to Svansson et al., 2009 [57], in brief: EHV-2 and EHV-5 were grown in primary kidney cells (prmEqFK) and RK-13 cells, respectively, until 90–100% cytopathic effect was observed. Cells were scraped off, washed in PBS, pelleted at 2000 x g, dissolved in lysis buffer and frozen at -80°C. Then thawed at 50°C and sonicated two times for 15 s on ice, the cell lysate spun at 48.200 x g for 20 min and the supernatant stored at -80°C and used as antigen in the ELISA. The negative control antigen was prepared in the same way from non-infected cells. MaxiSorp plates, 96-well flat bottom (Thermo Scientific), were coated with EHV-2, EHV-5 or negative control antigen, diluted 1:1000 in coating buffer (0.05 M carbonate-bicarbonate buffer, pH 9.5, Sigma-Aldrich), 100 μL/well. The ELISA was then performed according to Jonsdottir et al., 2018 [58], in brief: In each step 100 μL were added to the wells, except in the blocking step, where 200 μL were used. Serum and antibodies were diluted in blocking buffer (PBS containing 500 mM NaCl and 5% Tween 20 and 5% dried milk powder). Washing with high salt ELISA wash buffer (PBS containing 500 mM NaCl and 0.05% Tween 20) was done after each incubation step, 1 h at 37°C until addition of substrate. Non-specific binding sites were blocked with blocking buffer. The serum was diluted 1:100, added in duplicates and conjugate HRP-labelled Goat anti-horse IgG (Jackson ImmunoResearch) diluted 1:7000. The substrate, o-phenylenediamine dihydrochloride (OPD, Dako) and peroxide, was added and incubated in the dark for 10 min at RT. The reaction was stopped with 75 μL/well of 4NH_2_SO_4_ and optical density (OD) measured at 490 nm. For measurement of IgG subclasses, one step was added, antibodies against IgG1, IgG4/7 and IgG5 were used at a concentration of 1 μg/mL followed by HRP-labelled anti-mouse IgG (Jackson ImmunoResearch). Total IgG levels against EHV-5 were tested in a pilot experiment on serum from three foal/mare pairs (F/M no: 2, 9 and 13), from all time points sampled. The result was compared to the analogous EHV-2 test. The EHV-2 and EHV-5 curves between time points for each horse were parallel but all samples had lower IgG level against EHV-5 than against EHV-2. Based on these results, the EHV-2 antigen was used, and the ELISA interpreted as a γEHV ELISA. Total IgG and IgG1, IgG4/7 and IgG5 levels to the control antigen, were tested with serum from two foals (F no: 1 and 10) taken at 6 different time points, day 0 and months 1–4 and 12 and compared to comparable blank wells. No binding with the control was detected and the OD_490_ values were the same or lower than for the blank (OD_490_ < 0.1). Therefore it was not necessary to subtract the control, and wells with blank included on each plate for correction calculations.

Serum with a known titer was included on every plate in dilutions of 1:1600 (Total IgG), 1:100 (IgG1), 1:800 (IgG 4/7) and 1:50 (IgG5), in duplicate as standardization factor between plates. IgE antibody levels against EHV-2 were measured in ELISA according to Jonsdottir et al., 2015 [59].

### Virus isolation

Virus isolation was carried out in extEqFK cells (equine fetal kidney cells with extended life span) [60] in 24-well plates. Cells were propagated in Dulbecco’s MEM (DMEM, Thermo Fisher Scientific) supplemented with 2 mM glutamine, 100 U/mL penicillin, 100 μg streptomycin, 2% fetal bovine serum (FBS) (Thermo Fisher Scientific) and cultured at 37°C in humidified atmosphere with 5% CO_2_.

Virus isolation was performed from ten foal/mare pairs (F/M no: 1–4, 6, 10, 11–13 and 15) on all sampling points, except for day 5 where only three pairs were tested (F/M no: 2, 10 and 15) (S1 Table).

Virus isolation was attempted from NS and PBL samples, directly after sample collection, except at two months when only NS samples were tested after storage at -80°C prior to isolation. For PBL: 100 μL were added directly to the medium on confluent extEqFK cell layer. For NS: extEqFK cells were washed once with 200 μL of PBS, 200 μL of NS transport medium supernatant added to the cells and kept for 1 hr at culture conditions, aspirated and 1 mL of culture medium added.

Passage was done weekly for four weeks: The cell layer was scraped, and cells were centrifuged at 18.8 x g for 5 min and a new confluent cell layer was inoculated with 150 μL of the supernatant, for one hour at culture conditions. The remaining supernatant and cells were stored at -80°C. If cytopathic effects (CPE) were observed earlier, both cells and supernatant were harvested.

### DNA isolation

DNA was isolated from 120 μL BC with a Gentra Puregene Blood Core Kit (Qiagen). DNA from NS and from positive virus isolation was isolated from 200 μL supernatant with High Pure Viral Nucleic Acid Kit (Roche), according to manufacturer’s protocols.

### EHV-2 and EHV-5 type-specific quantitative Real-Time PCR

#### Cloning of glycoprotein B from EHV-2 and EHV-5

The complete glycoprotein B (gB) from EHV-2 and EHV-5 was amplified from strains EHV2-Bj and BB5-5 (GenBank accession no. HQ247738 and GQ325592, respectively) [61, 62] with Phusion Hot Start Flex 2x Master Mix (New England Biolabs) PCR according to manufacturer’s protocol. Primers are listed in S2 Table. A Fastbac HT B vector (Invitrogen) and the PCR amplicons were digested, for EHV-2 with endonucleases *Nco*I and *Spe*I (Thermo Scientific) and for the EHV-5 with *Eco*RI and *Not*I (Thermo Scientific), according to manufacturer’s DoubleDigestion calculator.

Dilutions of 10^3^–10^6^ copies per plasmid were used as positive controls, standard curve and for calculation of viral copy per 100 ng sample.

#### EHV-2 and EHV-5 specific quantitative real-time polymerase chain reaction (qPCR)

DNA isolated from NS, BC and positive virus cultures from foals and mares was tested for the presence of EHV-2 and EHV-5 with a qPCR assay according to Rushton *et al*. 2013 [19], targeting the gB gene (primers and probes are listed in S2 Table). 100 ng of DNA was used in each reaction, tested in duplicate. Positive controls were in triplicate on each plate and negative control (ddH_2_O) in duplicate. The reactions were performed in 96-well plates (Life Technologies) with AgPath-ID One-Step RT-PCR Kit master mix (Life Technologies), total reaction volume of 25 μL per well.

A Ct-value of 35 was chosen as the cut-off value for sample positivity for the positive virus cultures, based on qPCR results on extEqFK cells.

### Data analysis

The mares´ and foals´ ELISA results were analyzed for the whole group. According to the ELISA results the mares were then divided into two groups, depending on their total γEHV specific IgG values at day 0, low-mares (OD_490_ < 2.5, M no: 2, 5, 7, 10 and 12–15) and high-mares (OD_490_ >2.5, M no: 1, 3, 4, 6, 8, 9 and 11). The foals were then grouped according to their dams, in group-low (foals of dams with low total IgG at foaling) and in group-high (foals of dams with high total IgG at foaling).

Statistical analyses and graphs were performed with GraphPad Prism 6.0 (GraphPad Software). Each time point was examined separately for analysis of statistical difference between group-low and group-high, for the total IgG, IgG1 and IgG4/7 for foals and mares, and for the EHV-2 and EHV-5 viral load for NS. Total IgG and IgG4/7 values were normally distributed according to Shapiro-Wilk (p≥0.05) and analyzed with a two-tailed unpaired t-test (p<0.05); IgG1 values were not normally distributed and analyzed with a Mann-Whitney (p<0.05) test. EHV-2 and EHV-5 foal NS viral load data were not normally distributed and analyzed with a Mann-Whitney (p<0.05) test. The Kruskal-Wallis multiple comparison test was used to compare the time points internally for the EHV-2 and EHV-5 foals’ viral load for BC and NS, and Dunn’s test used to correct for multiple comparisons, p ≤ 0.05 was considered significant.

## Results

### Physical examination

During the observation period, only mild symptoms were occasionally noted in the foals. Mucopurulent nasal discharge was seen in three foals (F no: 3, 10 and 15) at two weeks of age. Mild keratoconjunctivitis was recorded in one foal (F3) at 4 months and in a majority of the foals when the flock was bled in December 2011, at 6 months. No clinical signs were seen in the mares during the 6-months observation period.

### γEHV specific antibody levels of the foal/mare pairs and comparison between foals with high or low maternal antibodies

#### γEHV specific antibody levels

**Fig 1:**
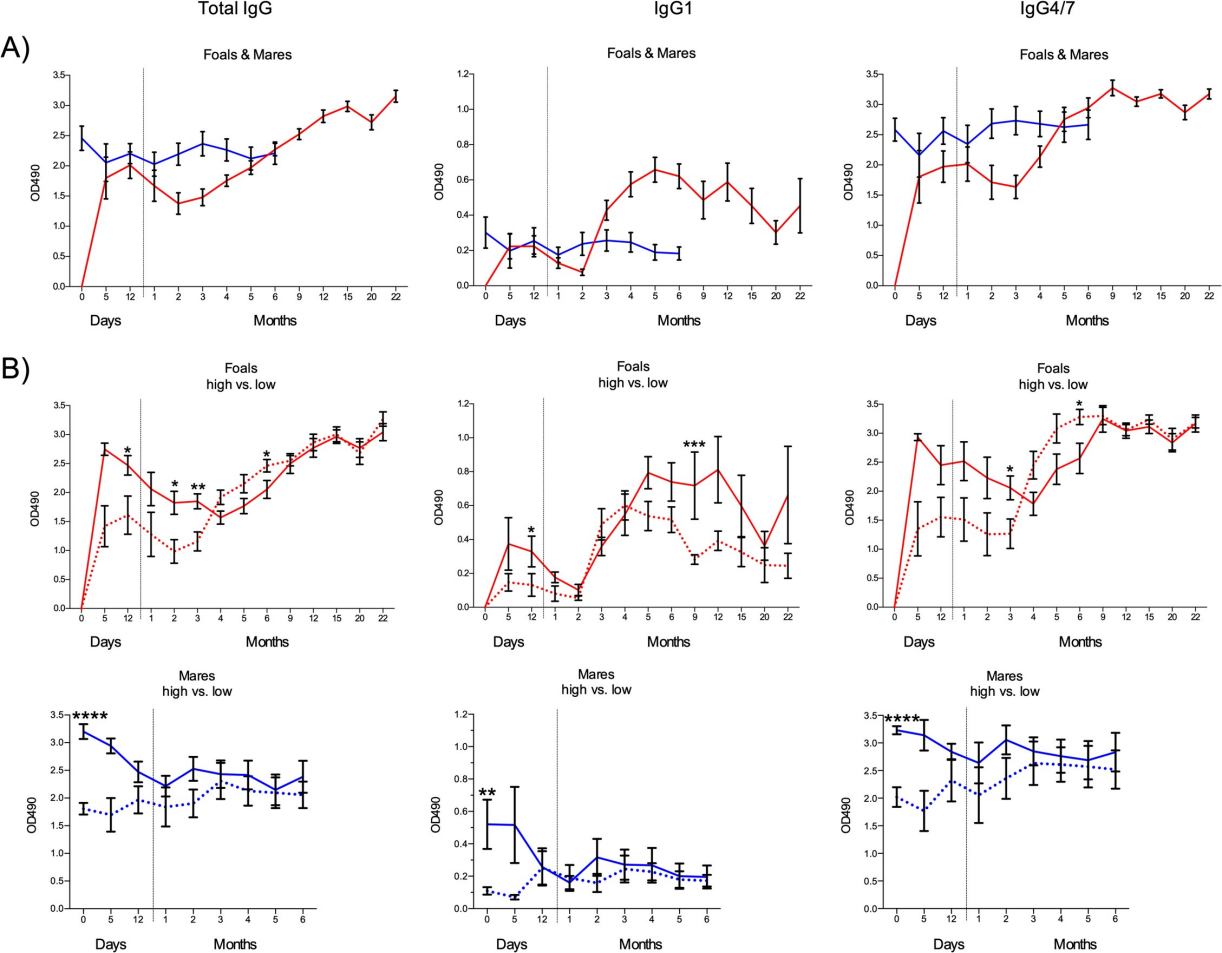
γEHV specific antibody response of foals and mares and comparison of γEHV total IgG response between foals with high versus low maternal antibodies, and between corresponding groups of mares. A) Total IgG, IgG1 and IgG4/7 in serum from 15 foals (red lines) and their dams (blue lines) over 22- and 6-month period, respectively. B) Group-high vs. group-low, for foals (red lines) and mares (blue lines), examined for total IgG, IgG1 and IgG4/7. The grouping was done according to the total γEHV IgG antibody level of the dams at foaling, group-high (solid lines, mares with OD_490_ values 2.7–3.8) and group-low (dotted lines, mares with OD_490_ values 1.5–2.2). Group-low: n = 6–8 and group-high: n = 6–7; except on day 5 where group-low: n = 5 and group-high: n = 2. Results are presented as mean ± SEM, two-tailed unpaired t-test for total IgG and IgG4/7 and Mann-Whitney for IgG1, *p≤ 0.05, **p<0.01, ***p<0.001, ****p<0.0001.

Pre-suckling sera from all 15 foals were negative for all γEHV antibodies tested. From day 12 on, γEHV specific total IgG levels in foals decreased gradually and reached a nadir at two months (Fig 1A). IgG levels then increased throughout the study, with the exception of month 20, where a small decrease was seen. IgG1 levels also decreased from day 12 and reached a nadir at 2 months, then increased and peaked at 5 months (Fig 1A). The levels then gradually decreased throughout the study with small fluctuations between time points. From month 1 the IgG4/7 levels decreased, nadir was at month 3, the levels peaked at month 9 and from then the levels were fairly stable throughout the study with minor fluctuations between time points (Fig 1A). The γEHV specific total IgG, IgG1 and IgG4/7 levels in the mare group varied between the mares but were relatively stable for each individual (Fig 1A).

The γEHV specific IgG5 and IgE levels were tested in serum from three foal/mare pairs, where the mares had low, medium and high total IgG level at day 0, pre-suckling, (F/M no: 2, 9 and 13). All were negative.

#### Comparison of γEHV IgG levels between foals of dams with high versus low maternal antibodies at foaling

The γEHV specific antibody levels of the mares varied considerably at foaling. Hence, for better understanding of the interconnection of antibody status and infection the mares were grouped into two groups, depending on their γEHV specific total IgG values at day 0, low-mares (OD_490_ < 2.5) and high-mares (OD_490_ > 2.5) (S1 Fig). Thus, total IgG, IgG1 and IgG4/7 at foaling were significantly different between these two groups (Fig 1B). The foals were then grouped according to their dams, in group-low (foals of dams with low total IgG at foaling) and in group-high (foals of dams with high total IgG at foaling).

Group-low foals had lower γEHV specific total IgG levels compared to group-high for the first 3 months, and this difference was significant at day 12 and at months 2 and 3 (Fig 1B). The antibody level gradually declined from day 5 for group-high and day 12 for group-low. Group-low reached nadir at month 2, and group-high at month 4. At months 4 to 6 group-low had higher levels of total IgG and this reached significance at month 6. From month 9 no difference was observed between the groups.

Group-low had lower γEHV specific IgG1 level for the first month, and this difference was significant at day 12 (Fig 1B). Both groups reached nadir at month 2 and there was little difference between the groups from month 2–4. From month 5 and throughout the study group-high had higher IgG1, significant at month 9.

The γEHV specific IgG4/7 levels in the foals showed the same pattern as the total IgG. Group-high had higher levels for the first 3 months and reached the lowest point at month 4 (Fig 1B). Group-low reached its nadir at month 3 and at that time point the difference between the groups was significant. At months 4 to 6 group-low surpassed group-high and was significantly higher at month 6. There was no difference between the groups from month 9 and throughout the study.

The γEHV specific total IgG, IgG1 and IgG4/7 antibody levels were significantly different between low-mares and high-mares at foaling / day 0 (Fig 1B). From that point the difference between the groups gradually decreased. The antibody levels for the high-mares overall decreased over the study period, but increased for the low-mares.

### EHV-2 and EHV-5 isolation from nasal swabs and blood from ten foal/mare pairs at different time points after birth

Virus isolation was attempted from 133 NS and 123 PBL samples from foals and 83 NS and 73 PBL samples from mares. The cultures showing CPE were tested with qPCR and they had either EHV-2 or a mixture of EHV-2 and EHV-5. Pure EHV-5 was not isolated.

**Fig 2:**
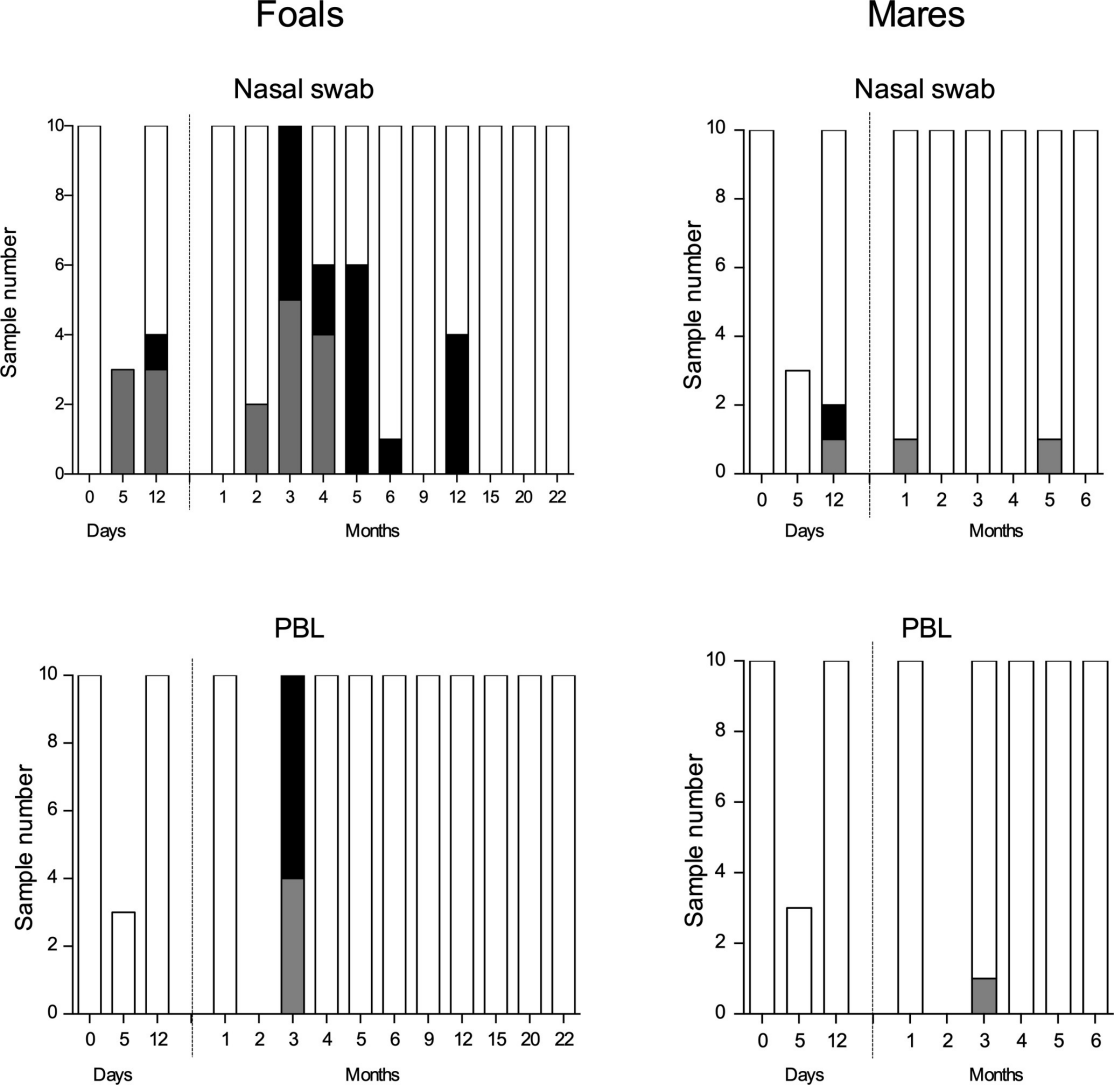
Isolation of γEHV from nasal swabs and PBL samples, from foals and mares. Virus isolation was attempted in extEqFK cells, from 10 foals and their dams over 22- and 6-month period, respectively. Cultures showing CPE were tested for EHV-2 and EHV-5 in specific qPCR, EHV-2 (gray columns), EHV-2 & EHV-5 (black columns), negative virus isolation (white columns).

Virus was most frequently isolated from the foal’s NS samples with a detectable CPE in 27% (36/133) of the cultures (Fig 2 and S3 Table). EHV-2 was first isolated on day 5. On day 12, overall EHV-2 had been isolated from 6/10 foals and EHV-5 from one foal that was also positive for EHV-2. For the first two months, all nine NS samples were EHV-2, except for the one mixture of EHV-2 and EHV-5, at day 12. At month two EHV-2 had been isolated overall from 7/10 foals. At three months, all foal NS samples were positive for either EHV-2 (50%) or a mixture of both viruses (50%). Similarly, PBL from foals were positive for EHV-2 (40%) or both viruses (60%). This was the only time point when virus was isolated from PBL of the foals. Between 5–12 months of ages, all positive cultures were double infections. Overall, EHV-5 was isolated from 8/10 foals during the first year and always together with EHV-2.

Virus was less frequently isolated from the mares. Only 4.8% (4/83) of the NS samples and 1.4% (1/73) of the PBL samples were positive (Fig 2 and S3 Table).

### EHV-2 and EHV-5 viral load in nasal swabs and blood from foals

A total of 198 NS and 199 BC samples from foals were tested for EHV-2 and EHV-5 DNA by qPCR. In NS, EHV-2 amplification was seen in 35% of the samples, EHV-5 alone was found in 2% of the samples, and 53.5% of the samples were positive for both viruses, while 9.5% of the samples were negative for both viruses. In the BC, amplification was seen in 43% of the samples for EHV-2 only, 1% for EHV-5 only, 54.5% positive for both viruses and no amplification in 1.5% of the samples.

**Fig 3:**
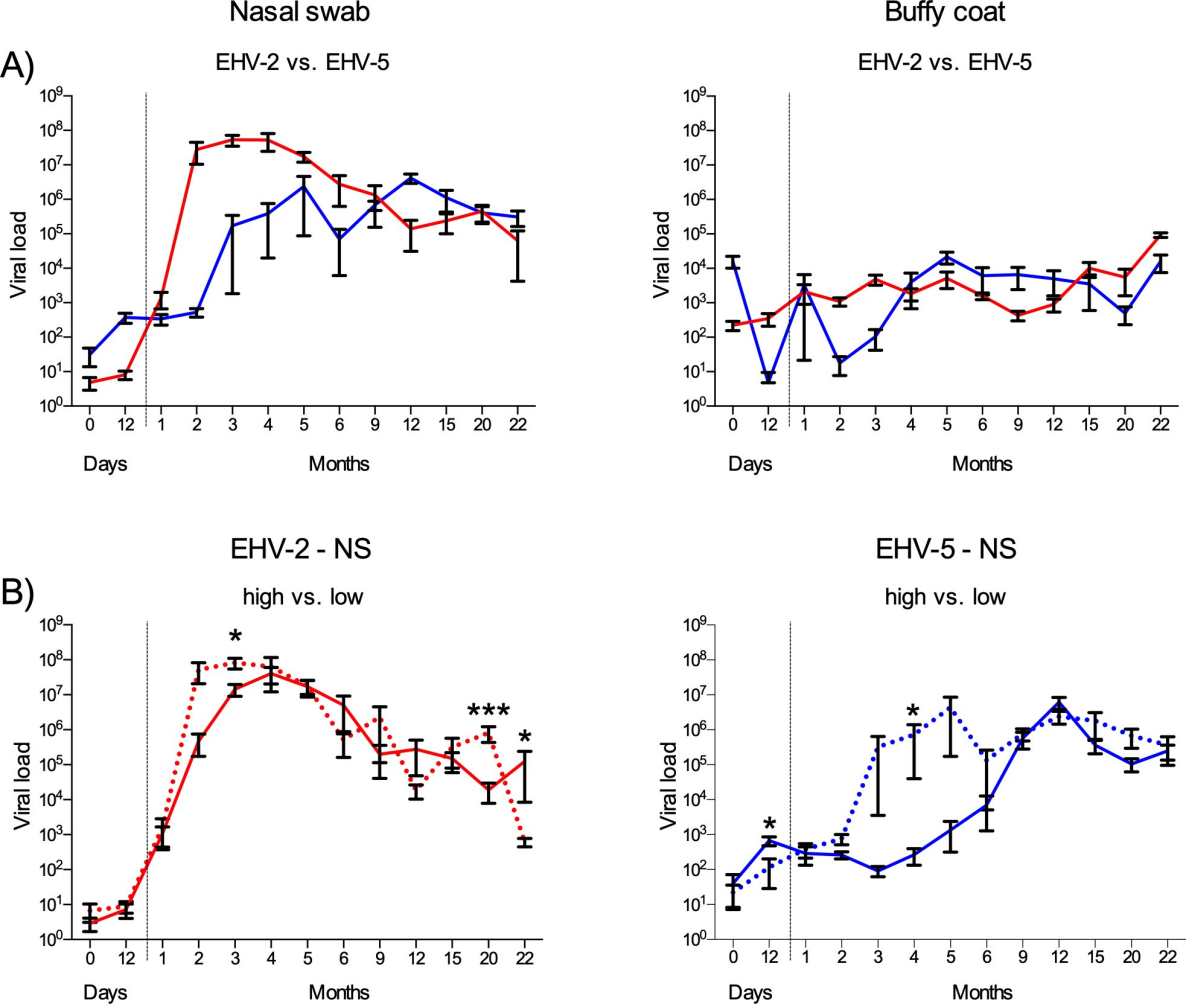
EHV-2 and EHV-5 viral load in nasal swab and buffy coat samples from the foals and comparison of γEHV viral load in nasal swab between foals with high versus low maternal antibodies. A) EHV-2 (red lines) and EHV-5 (blue lines) viral load measured with qPCR in nasal swab and buffy coat samples from 15 foals over 22-month period. B) Group-high (solid lines) vs. group-low (dotted lines), for EHV-2 and EHV-5 NS samples from foals. The grouping was done according to the total γEHV IgG antibody level of the dams at foaling, group-low (mares with OD_490_ 1.5–2.2) vs. group-high (mares with OD_490_ 2.7–3.8). Group-low: n = 7–8 and group-high: n = 6–7. The y-axis is log-scale. Viral load: Viral copy per 100 ng/DNA. Results are presented as mean ± SEM, Mann-Whitney, *p≤ 0.05, ***p<0.001.

The EHV-2 viral load in the NS samples increased only slightly for the first month, then rose significantly or almost 5-log and peaked at months 2 to 4 (Fig 3A). The load then decreased and was significantly lower at month 22 than months 3 to 5 (S4 Table). The EHV-5 viral load in the NS samples increased slowly for the first 5 months (Fig 3A). High individual variability was observed from month 3 to 6. The viral load had two peaks, at month 5 and a slightly higher peak at month 12. Month 12 was significantly higher than day 0 to month 6 (S4 Table). After month 12 the viral load decreased throughout the study.

The EHV-2 load in the BC increased slightly for the first 5 months (Fig 3A), then decreased and at month 9 was significantly lower than at months 3 and 5 (S4 Table). The viral load then increased and was significantly higher at month 15 than months 9 and 12, then decreased again around month 20, and the highest load was at month 22. Month 22 was significantly higher than other time points, except for months 3, 5 and 15. The EHV-5 viral load in the BC fluctuated for the first 2 months, then increased and remained constant from month 5 throughout the study (Fig 3A).

### Relation of the viral load to the dam’s γEHV specific total IgG antibody status at foaling

**Fig 4:**
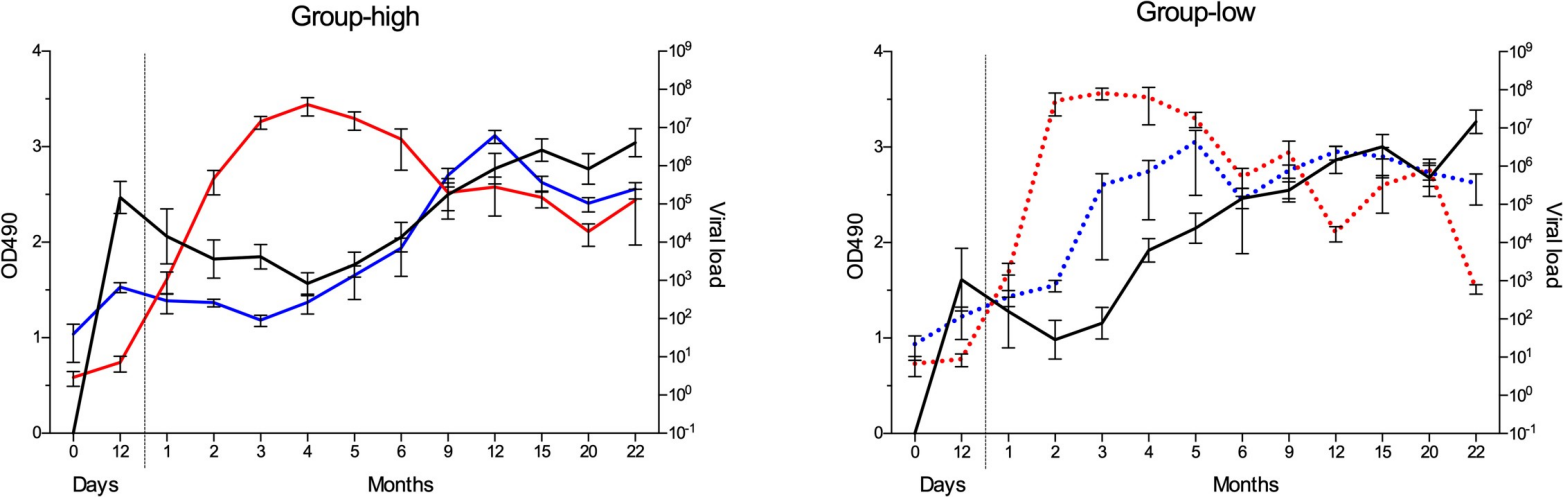
Comparison of total IgG γEHV specific antibody response and viral load in nasal swab samples from foals. The total IgG antibody level (black lined) in 15 foals over 22 month period was compared to the EHV-2 (red lines) and EHV-5 (blue lines) viral load, examined separately for group-high (solid lines) and group-low (dotted lines). The grouping was done according to the total γEHV IgG antibody level of the dams at foaling, group-low (mares with OD_490_ 1.5–2.2) vs. group-high (mares with OD_490_ 2.7–3.8). Results are presented at mean ± SEM. The right y-axis is log-scale. Viral load: Viral copy per 100 ng/DNA.

The viral load was examined in relation to the dam’s γEHV specific IgG level at foaling, group-low vs. group-high. A significant difference was seen between the groups in the EHV-2 NS at month 3 where group-low had a higher viral load (Fig 3B). In both groups the peak in the viral load was consistent with the decrease in maternal antibodies (Fig 4). For EHV-5, group-low had higher load at months 2 to 6, significantly at month 4 (Fig 3B). The group-high total IgG reached nadir at month 4, and 8 months later the EHV-5 viral load peaked, despite high endogenous production (Fig 4). In group-low the total IgG nadir was at month 2 and the viral load peaked at month 5, three months later, and the viral load remained relatively high throughout the study period (Fig 4).

## Discussion

The Icelandic horse is the only horse breed in Iceland. The horses were brought to the country in the 9^th^ and 10^th^ centuries and have been purebred since. Due to geographic isolation and a ban on import of live animals, they are immunologically naïve and free of many known severe horse pathogens, i.e. influenza, equine viral arteritis, *Rhodococcus equi*, strangles and EHV-1 [63]. In spite of this, horses in Iceland are known to be infected with the other four equine herpesviruses, EHV-2, EHV-3, EHV-4 and EHV-5, which in all likelihood were brought by the founders [7, 62].

The aim of the study was to determine the time-point of EHV-2 and EHV-5 infections and the time-kinetic of these viruses, to ascertain whether infection was influenced by the antibody status of the dam and to examine the influence of γEHV antibody status on viral load.

EHV-2 and EHV-5 can be recovered from horses at all ages, with and without clinical signs. This makes it difficult to define what role, if any, these viruses play in diseases. Only mild symptoms were noted in the foals on a few occasions, that could not be directly linked to γEHV infection, while no clinical signs were seen in the mares.

EHV-2 and EHV-5 have strong serological cross reactivity, as they share many common epitopes and cannot be distinguished by ELISA using whole virus antigens [1]. Therefore, the ELISA results are presented as γEHV specific. We did not detect γEHV specific antibodies in the foals’ serum before colostrum intake, supporting previous observations [15, 64].

As has been reported [53], there were two peaks in the antibody levels of the foals, first the maternal antibodies and the second when the endogenous production takes over. From day 12 the total γEHV specific IgG maternal antibody levels decreased and from month 3 the endogenous production dominated; the levels then continued to rise throughout the study. From month 1 the γEHV specific IgG4/7 maternal antibody levels decreased, and the endogenous production peaked at month 9. For IgG1 this was at day 12 and month 5, respectively.

The γEHV IgG1 antibodies of both foals and mares were low throughout the study period, while the IgG4/7 levels were comparable to the total IgG. We have previously reported the predominance of γEHV specific IgG4/7 over IgG1 for both foals and adults [57]. This has also been shown for the response to EHV-1 [65] and 4 [66], supporting that IgG4/7 is the long-lasting antibody isotype in equine herpes virus infections.

Many different biological factors can influence the transfer and uptake of the maternal antibodies, i.e. time of intake, concentration in colostrum and the amount transferred through the gut barrier. We grouped the foals according to their dam’s γEHV specific total IgG levels in serum at birth. It would have been optimal to base the grouping on serum from foals on day 4–5, when maternal uptake is over and endogenous production not started, but unfortunately we did not have samples from all the foals at that age. On day 12 the foals were infected with both EHV-2 and EHV-5 and the specific γEHV endogenous production had probably started. The foals in group-low had lower levels of maternal total IgG and IgG4/7 γEHV specific antibodies. Group-high had higher levels for the first 3 months, whereas group-low was higher at months 4 to 6, i.e. the endogenous antibody production was initially higher in group-low, probably because of earlier initiation of high viral replication. From month 9 onwards no difference was seen between the groups.

We assumed that group-low would peak in viral load sooner than group-high. This was true for the NS samples. Group-low had higher viral load at months 2–3 for EHV-2 and months 3–6 for EHV-5, significant at months 3 and 4, respectively. The antibodies from the mother seem to delay the infection and this was more evident for EHV-5 than for EHV-2.

When the total IgG response of the foals and the γEHV viral load in the nasal swabs were plotted together, separately for group-high and group-low, it was noted that the EHV-5 viral load somewhat synchronized to the endogenous antibody level over the sample period. This was seen for both group-high and group-low and could point towards antibody-dependent enhancement of the lytic infection [67, 68].

Previously we established fetal equine kidney cells with extended life span, extEqFK [60], which proved effective for infection with EHV-2 and EHV-5. EHV-5 has higher titer in the extEqFK cells compared to primary cells, indicating that these cells are well suited for EHV-5 isolation. Virus isolation was successful from NS of 5-day old foals. EHV-2 has to our knowledge never been isolated from such young foals. Initially we did not plan a virus isolation attempt at so early age, but when CPE was seen in samples from 12-day-old foals, virus isolation was attempted at day 5 from the three last-born foals and all the isolates were EHV-2. EHV-5 was first isolated at day 12 in a mixture with EHV-2. Virus isolation was only attempted from NS supernatants at month 2. The samples had been frozen before co-culture, which could have affected the number of virus isolations. Considering that the highest viral load of EHV-5 was at month twelve, we hoped for pure EHV-5 cultures at that time point, but no pure EHV-5 strains were isolated.

Only five virus isolations were acquired from the mares, 4 from NS samples and 1 from PBL. No viruses were isolated from foals older than one year. This could reflect a higher level and broader spectrum of neutralizing antibodies with age, although not measured directly in our γEHV ELISA [69, 70]. The virus isolation frequency was, however, lower than we have previously experienced. Using leukocyte enriched plasma for virus co-culturing is a time-saving method but it yields fewer cells than separating the leukocytes. In addition the plasma contains γEHV neutralizing antibodies, and together this could have made the virus isolation from PBL less efficient.

The general understanding is that the majority of foals are EHV-2 positive at 2–3 months of age, when the maternal antibodies decline [3, 12]. We confirmed these previous findings: the maternal γEHV IgG levels decreased in the foals at month 2 and were followed by a peak in EHV-2 viral load at months 2 to 4 in the NS. At month 3 γEHV was isolated from all foal samples, NS and PBL. Bell et al., 2006 [3] showed that foals are infected with the same EHV-2 strain as their dams during the first month, but later, when the foals are in more contact with other foals and horses, more types of EHV-2 strains are detected. This is presumably also the case in our study, as the low viral load detected in the foals in the first month is probably due to the effect of maternal antibodies directed to the same strains. When the maternal antibodies decline the foals become infected with more EHV-2 strains. Infection with EHV-5 has been reported to occur later than EHV-2 [5, 71] and our results support that, in more detail than has been shown before. The EHV-5 load in the NS rose more slowly and peaked 8–10 months later than EHV-2. This difference in viral load curves between EHV-2 and EHV-5 has not been explained but could be due to variation in replication rate and/or maturation of the subpopulation of target cells in the immune system.

With both PCR and qPCR methods both viruses have been detected from foals soon after birth, EHV-2 in nasal swab from 2-week-old foals [10] and in blood samples from 2- to 4-day-old foals [27] and EHV-5 in nasal swab 12–14 h after birth [3]. With virus isolation we detected both viruses in the foals in the first two weeks. Based on the viral load results, the age of foals when infected is harder to interpret, as amplification of EHV-2 and EHV-5 DNA was observed in a many of the day 0 samples. Higher viral load was seen in the positive day 0 BC samples than in NS. Using TaqMan master mix and probes did not alter this. We detected EHV-2 first in low number in NS of one foal at day 2 (35 viral copies/100 ng DNA), in BC from two foals at day 5 (2-10x10^3^ viral copies/100 ng DNA) and EHV-5 in NS at day 11 in two foals (7-14x10^2^ viral copies/100 ng DNA).

Both viruses are more frequently detected in young horses than in adults [17–19, 72] and this was also the case in our study. We tested in qPCR all NS from the mares for EHV-2 and EHV-5 (S2 Fig). During the first month the EHV-2 viral load in the NS was higher for the mares, but from then on, the foals had generally higher viral load. When the foals were one year old, the viral load was similar to the mares at month 6. The mares had higher EHV-5 viral load in the NS for the first 2 months, but after that the viral load was higher in the foals.

After transportation at month 22 the EHV-2 load rose uniformly in the BC for all foals and was significantly higher compared to the NS. It is known that stress can reactivate herpesviruses [73], and as the viruses are thought to be latent in B-lymphocytes [36] it is reasonable that the stress effects were not noted in the NS. A recent study, however, showed that shedding of EHV-2 increased significantly after transport [74], but viral load in blood was not measured.

## Conclusions

We have studied the time-kinetic of EHV-2 and EHV-5 infection in foals and the association between viral and antibody status of mare and foal. EHV-2 was first detected with qPCR 2 days after birth and first isolated at day 5; for EHV-5 this was day 11 and 12, respectively. EHV-5 viral load in the nasal swabs peaked 8–10 months later than EHV-2, or when the foals were one year old. The level of maternal antibodies had a notable effect on the viral load and endogenous antibody production.

## Supporting information

S1 Fig γEHV specific total IgG status of the mares at foaling. Group-low mares (n = 8) versus group-high mares (n = 7). The data were normally distributed according to Shapiro-Wilk (p≥0.05) and the results presented as mean, two-tailed unpaired t-test ****p<0.0001.
(TIF)

S2 Fig EHV-2 and EHV-5 viral load in nasal swab samples from the mares. A) EHV-2 and B) EHV-5 viral load measured in qPCR in nasal swabs from 15 mares over 6-month period. Viral load: viral copy per 100 ng/DNA. Results are presented as mean ± SEM.
(TIF)

S1 Table List of samples collected from each foal and mare over the study period. x: sample collected. -: sample not collected. **Bold**: used in virus isolation. F: foal. M: mare. Leuko: Leukocytes. NS: nasal swab. CA: chronological age.
(XLSX)

S2 Table Nucleotide sequence of primers, target genes and methods used. Location in glycoprotein B is based on strain BJ for EHV-2, GenBank no. HQ247738 and on strain BB5-5 for EHV-5, GenBank no. GQ325592.
(XLSX)

S3 Table Isolation of EHV-2 and EHV-5 from nasal swabs and PBL samples. Virus isolation with regard to specific foals and mares.
(XLSX)

S4 Table Comparison of γEHV viral load between different age groups within nasal swab and buffy coat samples. Kruskal-Wallis Multiple-Comparison Test was used to analyze the EHV-2 and EHV-5 viral load between age groups, within nasal swab and buffy coat samples.
(XLSX)
