# Supplementary figures and images for: The effect of maternal immunity on the equine gammaherpesvirus type 2 and 5 viral load and antibody response
Source: PLoS One. 2019 Jun 21;14(6):e0218576. doi: 10.1371/journal.pone.0218576 (PMC6588279; doi:10.1371/journal.pone.0218576)

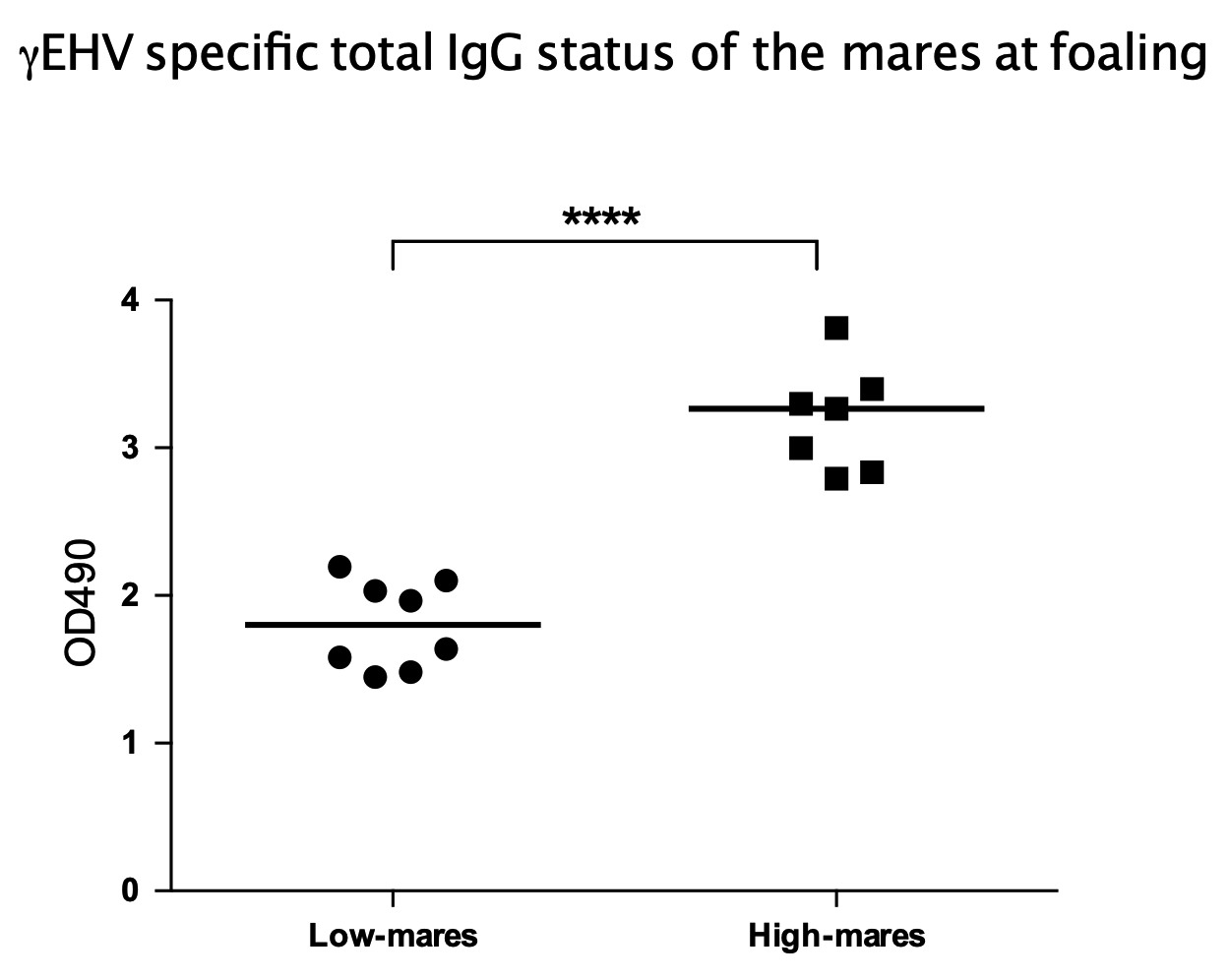

Supplement: S1 Fig — Group-low mares (n = 8) versus group-high mares (n = 7). The data were normally distributed according to Shapiro-Wilk (p≥0.05) and the results presented as mean, two-tailed unpaired t-test ****p<0.0001. (TIF) [file pone.0218576.s001.tif]

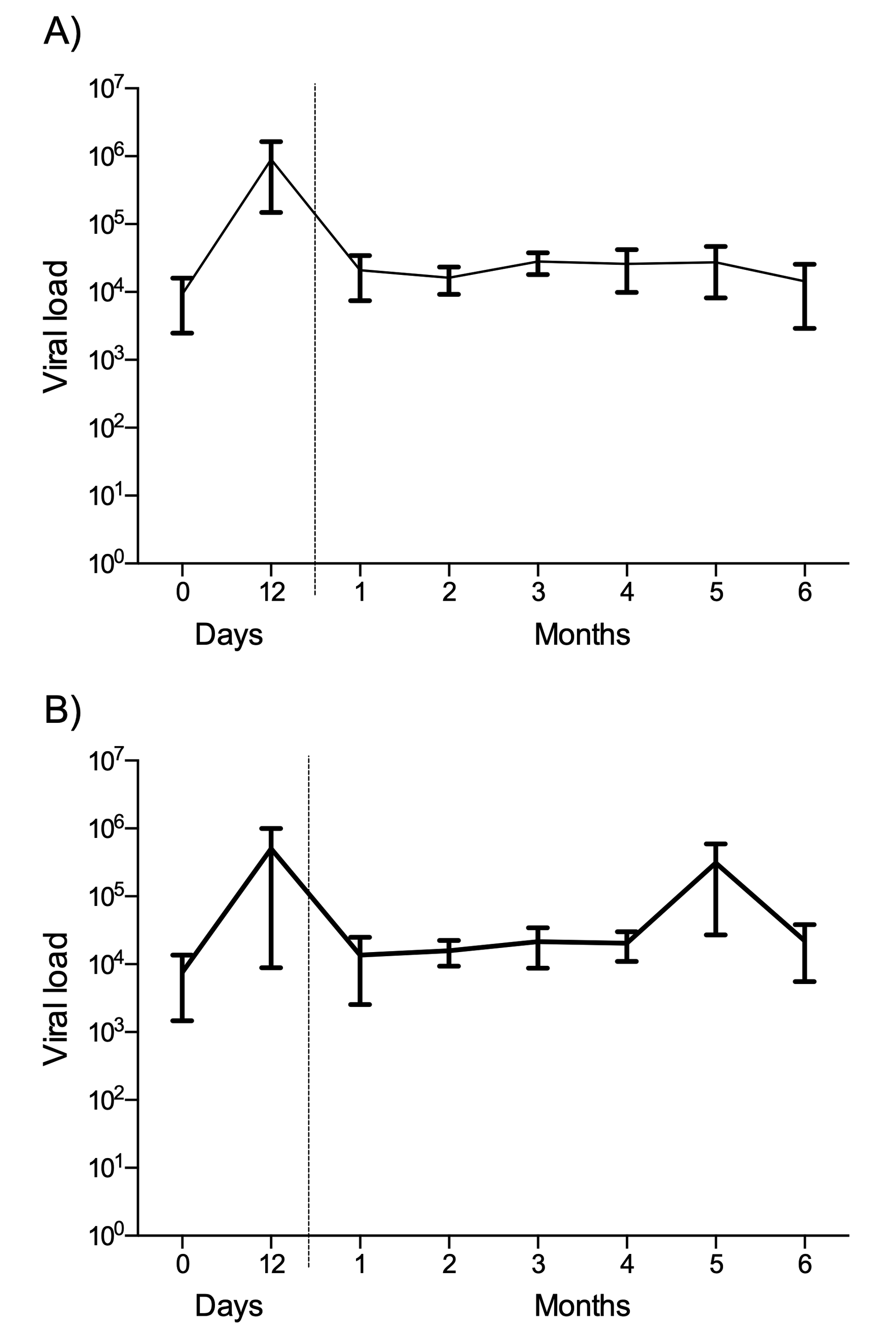

Supplement: S2 Fig — A) EHV-2 and B) EHV-5 viral load measured in qPCR in nasal swabs from 15 mares over 6-month period. Viral load: viral copy per 100 ng/DNA. Results are presented as mean ± SEM. (TIF) [file pone.0218576.s002.tif]
